# The release behavior and kinetic evaluation of tramadol HCl from chemically cross linked Ter polymeric hydrogels

**DOI:** 10.1186/2008-2231-21-10

**Published:** 2013-01-18

**Authors:** Muhammad A Malana, Rubab Zohra

**Affiliations:** 1 Chemistry Department, Bahauddin Zakarya University, Multan, Pakistan

**Keywords:** Tramadol HCl, Acrylic acid, Ter-polymer, Cross-linked, Release behavior

## Abstract

**Background and the purpose of the study:**

Hydrogels, being stimuli responsive are considered to be effective for targeted and sustained drug delivery. The main purpose for this work was to study the release behavior and kinetic evaluation of Tramadol HCl from chemically cross linked ter polymeric hydrogels.

**Methods:**

Ter-polymers of methacrylate, vinyl acetate and acrylic acid cross linked with ethylene glycol dimethacrylate (EGDMA) were prepared by free radical polymerization. The drug release rates, dynamic swelling behavior and pH sensitivity of hydrogels ranging in composition from 1-10 mol% EGDMA were studied. Tramadol HCl was used as model drug substance. The release behavior was investigated at pH 8 where all formulations exhibited non-Fickian diffusion mechanism.

**Results and major conclusion:**

Absorbency was found to be more than 99% indicating good drug loading capability of these hydrogels towards the selected drug substance. Formulations designed with increasing amounts of EGDMA had a decreased equilibrium media content as well as media penetrating velocity and thus exhibited a slower drug release rate. Fitting of release data to different kinetic models indicate that the kinetic order shifts from the first to zero order as the concentration of drug was increased in the medium, showing gradual independency of drug release towards its concentration. Formulations with low drug content showed best fitness with Higuchi model whereas those with higher concentration of drug followed Hixson-Crowell model with better correlation values indicating that the drug release from these formulations depends more on change in surface area and diameter of tablets than that on concentration of the drug. Release exponent (n) derived from Korse-Meyer Peppas equation implied that the release of Tramadol HCl from these formulations was generally non-Fickian (n > 0.5 > 1) showing swelling controlled mechanism. The mechanical strength and controlled release capability of the systems indicate that these co-polymeric hydrogels have a great potential to be used as colon drug delivery device through oral administration.

## Introduction

Tramadol, a synthetic opioid of the amino-cyclohexanol group, exhibiting weak opioid agonist properties, is a centrally acting analgesic and has been observed to be effective in both experimental and clinical pain and additionally causes no serious cardiovascular or respiratory side effects [1]. The usual oral dosage requirement of the drug is 50 to 100 mg every 4 to 6 hours with a maximum dosage of 400 mg per day [2]. A sustained-release formulation of tramadol is required to improve patient compliance and to reduce the administration frequency. To modulate the drug release, the most commonly used method is to include it in a matrix system [3]. As hydrophilic polymer matrix systems are proved to be flexible to obtain a desirable drug release profile, these are widely used in oral controlled drug delivery [4]. Using a hydrophilic matrix system to release the drug for extended duration, especially, for highly water-soluble drugs, is restricted to rapid diffusion of the dissolved drug through the hydrophilic gel structures. For such highly soluble drug substances, chemically cross linked hydrogels are considered suitable as matrixing agents for developing sustained-release dosage forms. This extensive use corresponds to the non-toxicity, high drug loading capacity and pH-sensitivity of the network structure. Moreover, a problem, frequently, encountered with the oral administration of these formulations, is inability to increase the resistance time in stomach and proximal portion of small intestine [5]. To overcome this problem, it becomes necessary to prolong the dosage form either in the stomach or somewhere in the upper small intestine until all the drug is released over the desired period of time [6,7].

These polymer matrices can be either chemically cross linked having covalent bonding or physically cross linked through hydrogen bonds depending on the monomers, polymerization methods and the mode of application [8]. Advance research has flashed over synthesizing and characterizing hydrogels having particular mechanical properties such as strength and modulus, environmental sensitivity to temperature, electric field, pH or ionic strength and mass transport control that can be “tuned” to get special pharmacological application including reduced toxic “burst” effects of a drug, protections of fragile drugs in their dosage environment and location specific dosage etc. [9].

In the present work, the main objective was to study the release behavior and kinetic evaluation of a model drug tramadol HCl from chemically cross linked (Vinylacetate-co-methacrylate-co-acrylic acid) (VA-co-MA-co-AA) co-polymeric ternary systems. The preliminary swelling studies [10] are indicative of the fact that these hydrogels may be able to face the problems related to sustained drug delivery. For this purpose chemically cross linked co-polymeric hydrogels were prepared with a range of cross linker concentration. Effect of the concentration of the cross linker on various swelling parameters and the drug release profiles was estimated. Influence of amount of drug loaded on drug release rate was also studied in detail. Different release models were applied to evaluate the release kinetics of the drug from the optimized formulation.

## Experimental

### Materials and polymer preparation

Two hydrophobic monomers, methacrylate (MA) (MERCK, 99%) and vinylacetate (VA) 9Fluka, 99%) were mixed with a hydrophilic monomer, acrylic acid (AA) (fluka, 99%) to prepare the polymer for this study. Copolymers of MA, VA and AA were prepared according to the previously reported method by free radical polymerization [10] using Benzoylperoxide (BPO) (MERCK, 100%) as the initiator and ethanol as solvent. Different grades (E_1_, E_2_, E_3_, E_4_ having 1, 3.5, 6.5, 10 mol% respectively) of the cross linker Ethylene glycoldimethacrylate (EGDMA) (Fluka, 100%) were added to prepare chemically cross linked co-polymeric networks.

### Swelling studies

The dried hydrogel disks were immersed in excess of the swelling medium (50ml) having a pH range from 1-8 at 37°C. At regular intervals of time, the disks were removed from the solution and weighed after excess solution on the surface was blotted. The experiment was performed in triplicate and the swelling parameters like media penetration velocity (ν), equilibrium media content (Q_e_) and diffusion exponent were calculated using following equations [10]:

(1) $$\text{\%S}\;=\;m_{t}{-m}_{o}{/m}_{o}\;\times 100$$

Where m_t_ is the mass of the swollen hydrogel and m_o_ is the mass of xerogel.

(2) $$1n\;W_{t}/W_{e}\;=\;1\mathrm{nk}\;+\;n\;1\mathrm{nt}$$

Where W_t_ is the dynamic media content at time t and W_e_ is the equilibrium media content, n stands for diffusion exponent and k is the rate constant.

### Drug loading

The dried polymer disks were loaded with Tramadol HCl by soaking them in various drug solutions (T_1_, T_2_, T_3_, T_4_, T_5_, T_6_ having 0.8, 1.6, 2.4, 3.2, 4.0, 4.8 mg/ml respectively) in phosphate buffer solutions of pH 8.0 at 37°C till the equilibrium swelling was attained. This method was preferred to in situ drug loading to avoid any probable degradation of the drug substance or undesirable drug-polymer reaction when high temperature was applied during the synthesis process. The wet drug loaded polymers were dried at room temperature through simple evaporation. The drug loaded dried polymer disks were cloudy when compared to similar disks without the drug. The absorbency of tramadol by the hydrogels was calculated using the following equation [11]:

(3) $$\mathrm{Absorbency}\;\left(Q\right)\;=\;\left(C_{1}V_{1}{-C}_{2}V_{2}\right)/m_{o}$$

Where Q (mg/g) is the absorbency of tramadol by the xerogel; C_1_ (mg/ml) is the initial concentration of tramadol solution; V_1_ (ml) represents the initial volume of tramadol solution; C_2_ (mg/ml) is the concentration of tramdol after absorption by the xerogel; V_2_ (ml) is the volume of tramadol solution after absorption by the polymer; and m_o_ is the mass of the polymer in dry state.

### In vitro drug release studies

In vitro drug release of Tramadol HCl from co-polymeric hydrogels was evaluated in triplicate using UV-Visible spectrophotometry. The dried drug loaded disks were transferred into the fixed volume of buffer solution of pH 8 at 37°C. At specified time intervals, 3 ml of aliquots were removed from every buffer solution and the absorbance was noted using UV-visible spectrophotometer at the maximum absorption wave length (240nm) already measured using a stock solution of Tramadol HCl in phosphate buffer of pH 8. Three aliquots of various solutions were studied for any single point of release curve. After absorbance measurements, aliquots were returned to the original solution, so that the volume may be kept constant. To transform absorbance determinations into concentrations, calibration curve was used [12].

### Release kinetics

To study the release kinetics of Tramadol HCl from the matrix tablets, the release data were fitted to the following equations:

Zero order equation [13]:

(4) $$Q_{t}=k_{o}\;t$$

Where Q_t_ stands for the percentage of drug released at time t and k_o_ is the release rate constant;

First order equation [14]:

(5) $$1n\;\left(100-\;Q_{t}\right)\;=1n100-k_{1}t$$

Where k_1_ stands for release rate constant for the first order kinetics;

Higuchi’s equation [15]:

(6) $$Q_{t}=k_{H}\;t^{1/2}$$

Where k_H_ represents the Higuchi release rate constant;

Hixson-Crowell model [16]:

(7) $${\left(100-\;Q_{t}\right)}^{1/2}=100^{1/3}-k_{\mathrm{HC}}\;t$$

Where, k_HC_ stands for Hixson-Crowell rate constant.

Moreover, for better characterization of the drug release mechanisms, the Korsmeyer-Peppas [17] semi-empirical model was applied:

(8) $$Q_{t}/Q_{e}=k_{\mathrm{KP}}\;t^{n}$$

Where Q_t_/Q_e_ is the fraction of the drug released at time t, k_KP_ is a constant corresponding to the structural and geometric characteristics of the device and n is the release exponent which is indicative of the mechanism of the drug release. In case of cylindrical geometries such as tablets, for fitting the data to the equations, only the points within the interval 10-70% were used. In case of Hixson-Crowell and korsmeyer-Peppas models, the data taken was within 10-60% drug release.

### Mechanical strength

Mechanical strength of dried as well as hydrogels swollen at different pH values up to equilibrium point was determined applying the weight on them until the hydrogels were fractured [18,19].

### DSC/TGA analysis

Thermal degradation of the hydrogel systems was studied using a thermo-gravimetric analyzer [TA Instruments SDT Q. 600 V20 .9 Build 20 simultaneous TGA-DSC], by heating them from room temperature to 600°C at a heating rate of 10°C/min under a nitrogen flow.

## Discussion

### Media penetration velocity and equilibrium media content

**Figure 1:**
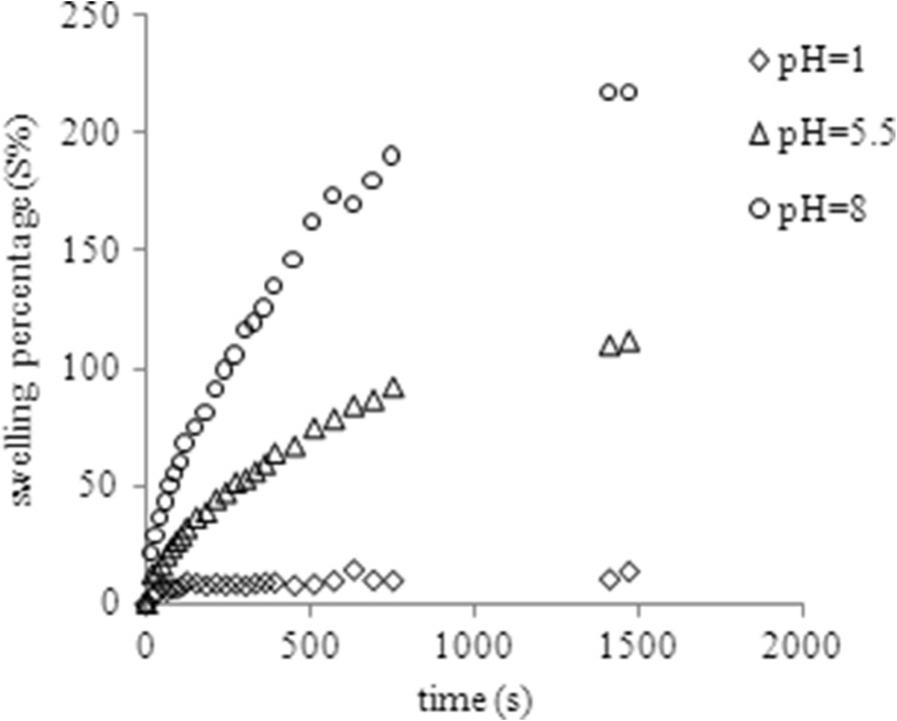
Media sorption profile for optimized concentration of the cross linker (3.5 mol%) exhibting effect of pH.

**Figure 2:**
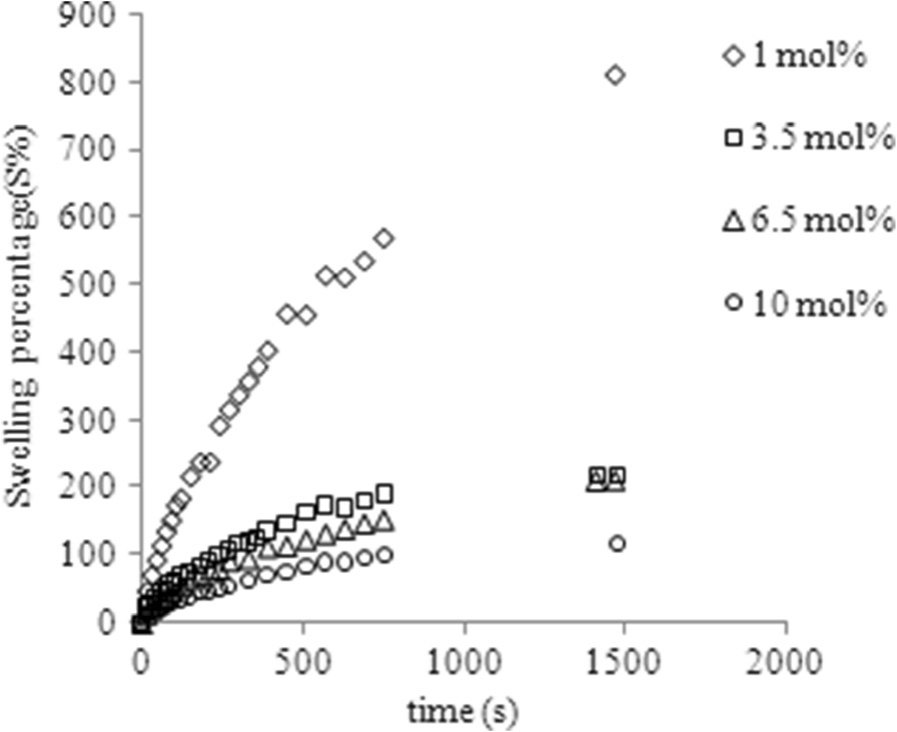
Media sorption profile at pH 8 for copolymers comprised of 1-10 mol% EGDM.

Figures 1 and 2 show the influence of the copolymer composition and the media pH on the dynamic swelling behavior of the copolymers. As the concentration of EGDMA in the polymer network increased, the rate of sorption and equilibrium water content was decreased. This is not surprising since AA is hydrophilic and EGDMA binds AA to increase the cross link density in the hydrogels which lowers the average molecular weight between the cross links and this curtails the free volumes accessible to the penetrant water molecules [18]. As far as pH of the medium is concerned, the highest swelling rate and equilibrium media content were observed at pH 8. It has already been reported that acidic hydrogels show greater media sorption rate in basic medium where most of the free carboxylic groups get ionized to produce negatively charged carboxylate ions which cause repulsions ultimately resulting in higher swelling rate as well as equilibrium media content [20].

The rate of advancement of glassy to rubbery front from the surface to the center of the polymer disk was determined by calculating the media penetration velocity (ν),

(9) v=1/2ρAδw/δt

**Figure 3:**
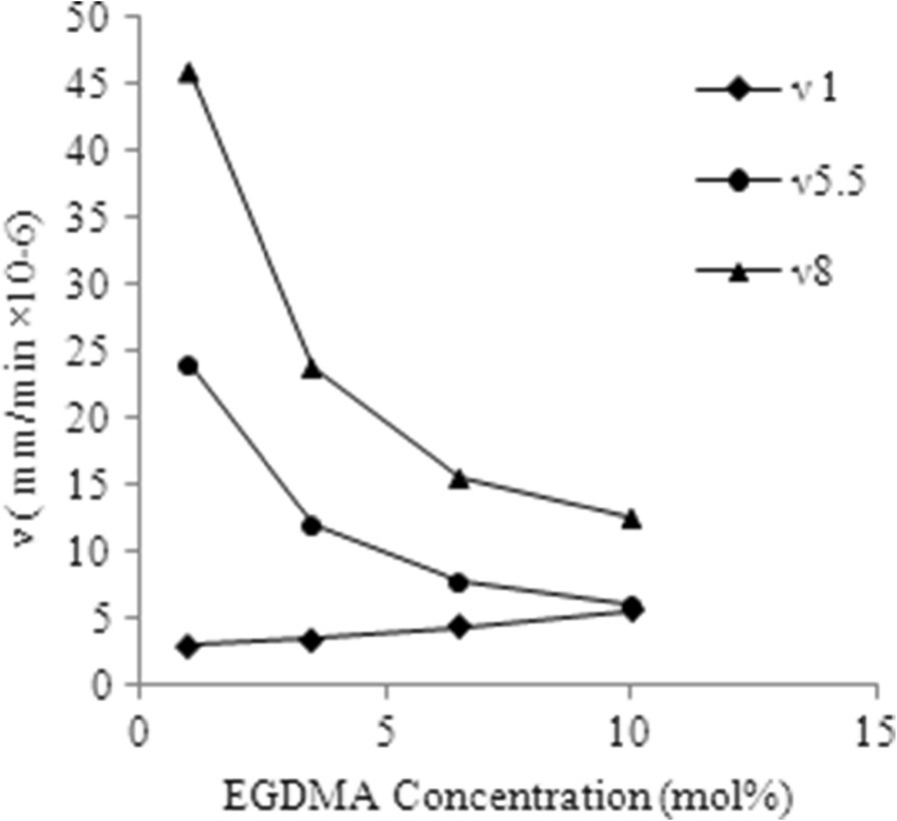
Media penetration velocity at pH 1-8 in polymers comprised of 1-10 mol% EGDMA.

Where “ρ” is the density of the medium, “A” represents the area of the one disk face, “w” is the mass gained by the polymer and “t” is the time. The early time data (t<15 min) were used to calculate the media penetration velocity. It has been reported, as the media penetrates the glassy polymer, the solvent swells the polymer and produces a rubbery region [21]. Figure 3 shows that the media penetration velocity decreased by 75% at pH 5.5 and 73% at pH8 and on the other hand the equilibrium media content also decreased by an order of magnitude as the concentration of EGDMA was increased from 1-10 mol % at all the pH values. A similar degree of decrease in media penetration velocity was observed in other hydrophobic-hydrophilic copolymers like poly (HEMA-co-MMA) as the proportion of hydrophobic monomer MMA was increased from 0-40% [21,22].

The change in media penetration velocity with increasing EGDMA content may be due to two possible mechanisms: first if the media travelled primarily through the hydrophilic AA regions of these copolymers, the increasing number of cross link domains might keep AA content busy in building the cross links that could obstruct the media diffusive path way for diffusion; secondly, the increased cross linker’s concentration may also inhibit polymer chain relaxation, thus reducing the free volume through which the media can travel [23].

**Figure 4:**
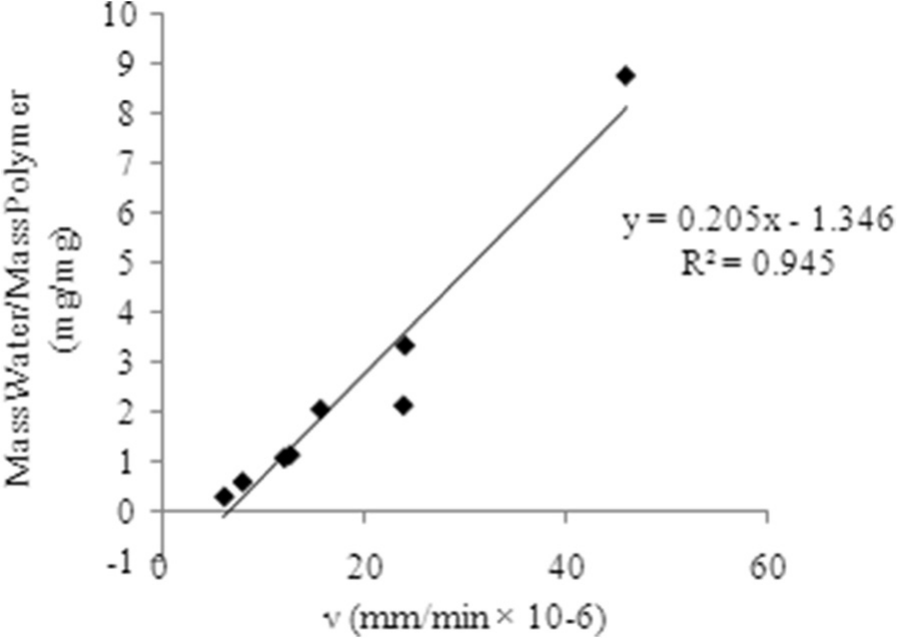
**Equilibrium media content at pH higher than pK**_**a**_**of AA as a function of media penetration velocity in the polymers comprised of 1-10 mol% EGDMA.**

However, the media penetration velocity increased (46.6%) when the concentration of EGDMA was increased at pH=1. It is assumed that pK_a_ value of AA (4.75) appeared to have a great impact on penetration velocity. It was concluded that the equilibrium media content was directly proportional to the media penetration velocity at the pH higher than pK_a_ value of AA (Figure 4). This trend has also been observed in poly (NIPA-co-FOSA) copolymers [24]. This relationship is also important because it suggests that from the media penetration velocity, the equilibrium media content can be predicted in very short experimental time (i.e on the order of minutes vs hours or days) at a specific pH. But below pK_a_ value the inverse relationship was observed between the media penetration velocity and equilibrium media content. The most probable reason for this unexpected behavior for these polymeric systems may be that the carbonyl oxygen atom of the EGDMA present on the surface of disks should facilitate the intermolecular hydrogen bonds with surrounding water molecules during early times of exposure of hydrogels. However, as a continuous column of water is developed from outside to inside of the disk, the increased crosslink density predominates to cause a usual decrease in equilibrium media content. The formation of hydrogen bonds by carbonyl oxygen with water molecules contributing an increase in media penetration velocity has also been suggested by Hajimi et al. [25].

### Media diffusion mechanism

The mechanism of the media sorption was estimated by calculating the diffusion exponent (n) using Korsmeyer-Peppas model [10]. The values are tabulated in Table 1. It was found that all formulations followed Fickian mode of media sorption (values of “n” are ranging from 0.100 to 0.407) at pH below pK_a_ value of AA content, whereas the values of “n” were greater than 0.5 above pK_a_. It was observed that increased pH values shifts the mechanism from diffusion-controlled to an anomalous transport in which both the concentration gradient and erosion are governing the diffusion mechanism. It is suggested that the polymer matrix maintains its structure in acidic conditions and the media sorption is mainly controlled by diffusion, whereas the polymer chains get relaxed in the basic media. It has been reported that in the anionic hydrogels like having carboxylic groups attached with the polymeric chains, the H^+^ ions can combine with OH^-^ ions present in the basic solution to produce water. The cations joined with other hydroxyl groups, may compensate the charge, going into the polymeric network, thus leading to an osmotic pressure increase which is responsible for the swelling of the hydrogels [11]. At swelling equilibrium, the recovery elastic force is equal to the osmotic pressure [26,27].

**Table 1:** Summary of media penetration velocities, equilibrium media contents, mechanical strength and power law parameters for the polymers

**Sample**	**Media penetration velocity**	**Equilibrium media sorbed**	**Mechanical strength**	**Fick’s model**	
	**(mm/min×10**^**-6**^**)**	**(mg media/mg polymer)**	**(gm)**	**n**	**R**^**2**^
**pH =1**					
E_1_	3.027	0.1923		0.407	0.965
E_2_	3.47	0.1336		0.290	0.925
E_3_	4.46	0.0782		0.189	0.911
E_4_	5.67	0.06923		0.206	0.721
**pH =5.5**					
E_1_	24	3.713		0.671	0.996
E_2_	12	1.114		0.557	0.976
E_3_	7.8	0.630		0.558	0.976
E_4_	6	0.3342		0.507	0.991
**pH =8**					
E_1_	45.99	8.79	452	0.662	0.999
E_2_	23.85	8.79	490	0.572	0.996
E_3_	15.6	2.085	560	0.603	0.996
E_4_	12.6	1.173	634	0.571	0.992

### In vitro release of tramadol HCl from the copolymer

**Figure 5:**
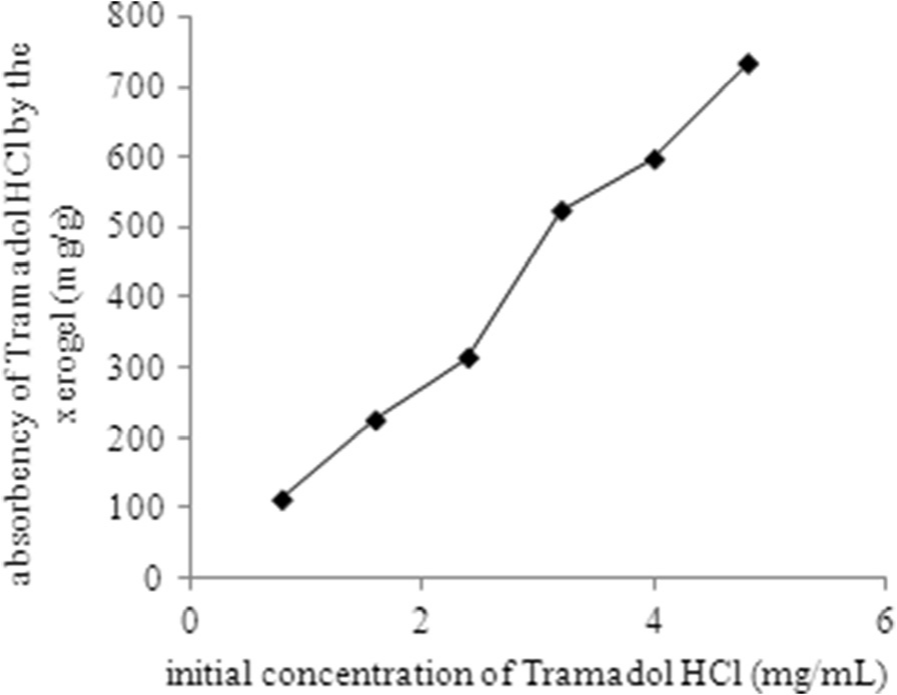
Absorbency of Tramadol HCl by the xerogel having 3.5 mol% EGDMA provided with various initial concentrations of the drug.

Owing to preliminary swelling studies, the drug loading and release studies were carried out at pH 8 and 37°C where all the formulations attained the highest rate of sorption and equilibrium media content. Figure 5 exhibits the absorbency of Tramadol HCl by the xerogel with different EGDMA content as well as drug concentration. As the mol% of the cross linker in the copolymer hydrogels increases, less tramadol was absorbed because of low water up take. The amount of the drug loaded in the hydrogel has a close relation with the cross link density of the hydrogels. As the increased concentration of the cross linker increases the cross link density, so the amount of tramadol loaded decreases as also reported by other authors [28].

**Figure 6:**
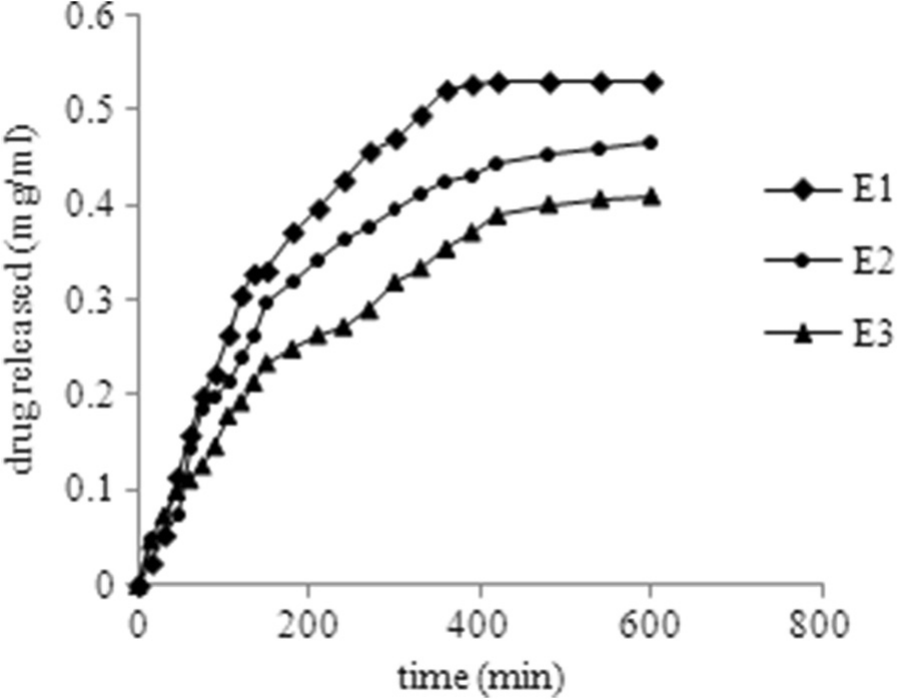
Influence of amount of Tramadol HCl in the matrix on the release rate for 3.5 mol% EGDMA at pH 8.

**Figure 7:**
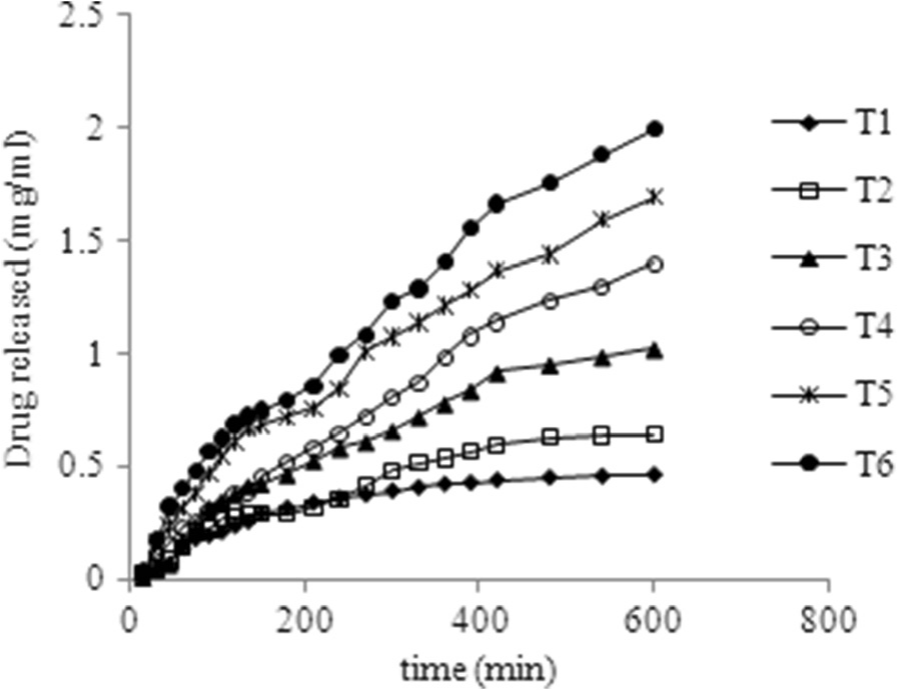
Influence of the concentration of the cross linking agent on the release rate of Tramadol HCl.

Drug release studies were performed with respect to the concentration of the cross linker (Figure 6) and the drug content available to be released by the system (Figure 7), because it has been reported that the chemical structure and dissolution in water do not show a significant influence on the drug release rate from the hydrogel networks; on the other hand the cross link density and the amount of the drug loaded determine the drug release from the system [29]. The drug release data are shown in the Table 2.

**Table 2:** Kinetic parameters of tramadol HCl release from the matrix tablets

**Formulation**	**Zero-order**		**First-order**		**Higuchi**		**Hixson-Crowell**		**Korsmeyer-Peppas**		
	**k**_**o**_**(%min**^**-1**^**)**	**R**^**2**^	**k**_**1**_**(min**^**-1**^**)**	**R**^**2**^	**k**_**H**_**(%min**^**-1/2**^**)**	**R**^**2**^	**k**_**HC **_**(%min**^**-1**^**)**	**R**^**2**^	**n**	**R**^**2**^	**k**_**KP**_** (%min**^**-n**^**)**
E_1_	0.339	0.957	0.007	0.990	7.715	0.989	0.009	0.965	0.907	0.987	7.15
E_2_	0.332	0.941	0.006	0.987	6.68	0.980	0.008	0.974	0.982	0.893	4.52
E_3_	0.23	0.942	0.004	0.981	4.921	0.983	0.005	0.974	0.691	0.990	16.0
T1	0.332	0.941	0.006	0.987	6.83	0.980	0.008	o.974	0.982	0.893	4.52
T2	0.131	0.963	0.002	0.973	3.803	0.967	0.003	0.949	0.713	0.914	9.18
T3	0.123	0.978	0.002	0.986	3.827	0.989	0.002	0.984	0.748	0.969	7.019
T4	0.146	0.996	0.002	0.973	4.309	0.978	0.003	0.990	0.832	0.994	4.13
T5	0.096	0.991	0.001	0.989	3.155	0.983	0.002	0.990	0.688	0.988	7.99
T6	0.0127	0.985	0.002	0.989	3.701	0.989	0.002	0.991	0.733	0.979	7.73

To analyze the effect of the concentration of the cross linker on the release behavior of the drug, only three samples E_1_, E_2_ and E_3_ were used for experiment as the sample E_4_ was collapsed during the loading process (Figure 6). The most probable explanation for the behavior of E_4_ may be that there is some type of repulsive interaction between the material of the drug loaded and the highly cross linked dense polymeric matrix. Figure 6 is showing the release profile for influence of the cross linking agent concentration. As predicted from swelling studies, the drug release rate decreased with increase in the cross link density. Same effect of the cross linker’s concentration on the drug release rate has also been reported by other authors [20]. On the other hand, the release rate was enhanced by the amount of the drug loaded in the polymer networks. The optimized formulation (E_2_) was used to study the effect of amount of the drug loaded in the system as shown in the Figure 7.

In initial stages the effect was not pronounced. The difference was negligible up to 1.6 mg/ml initially prepared drug concentration solution. However, for higher drug loading concentrations, the amount of the drug release as well as the drug release rate were increased significantly. The root cause for the observed effect might be the higher concentration gradient which is responsible for a more efficient diffusion of the drug substance through the polymer network, keeping all other conditions the same. Hence, variation in drug loading concentration offers a real probability of controlling the drug release [30].

Various mathematical equations have been proposed to describe the kinetics of the drug release from the controlled release formulations. The zero order model equation (Eq. 4) describes the systems, where the drug release does not depend on its concentration [13]. The first order release kinetics describes the dependency on the drug concentration in the polymeric networks [14]. Higuchi model proposes a direct relation of the drug release from the matrix to a square root of time and is based on the Fickian diffusion [15]. The Hixson-Crowell cube root law describes the release rate from the systems depending on the change in surface area and diameter of the particles or tablets and specifically is applied for the systems which erode over time [16].

**Figure 8:**
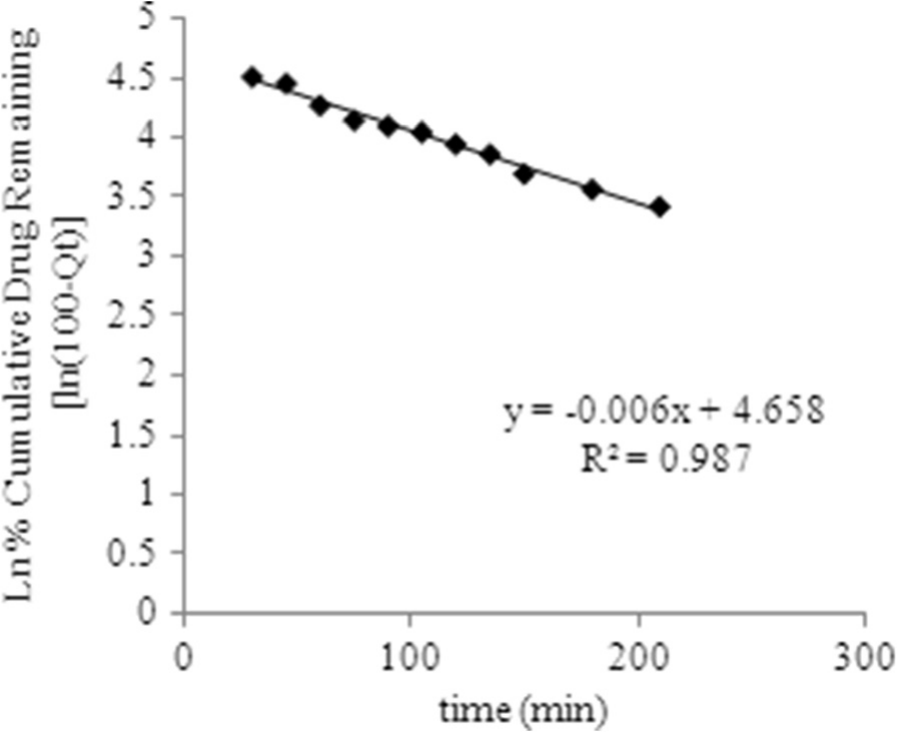
**First order release kinetics of optimized formulation E**_**2**_**having drug concentration 0.8 mg/ mL.**

**Figure 9:**
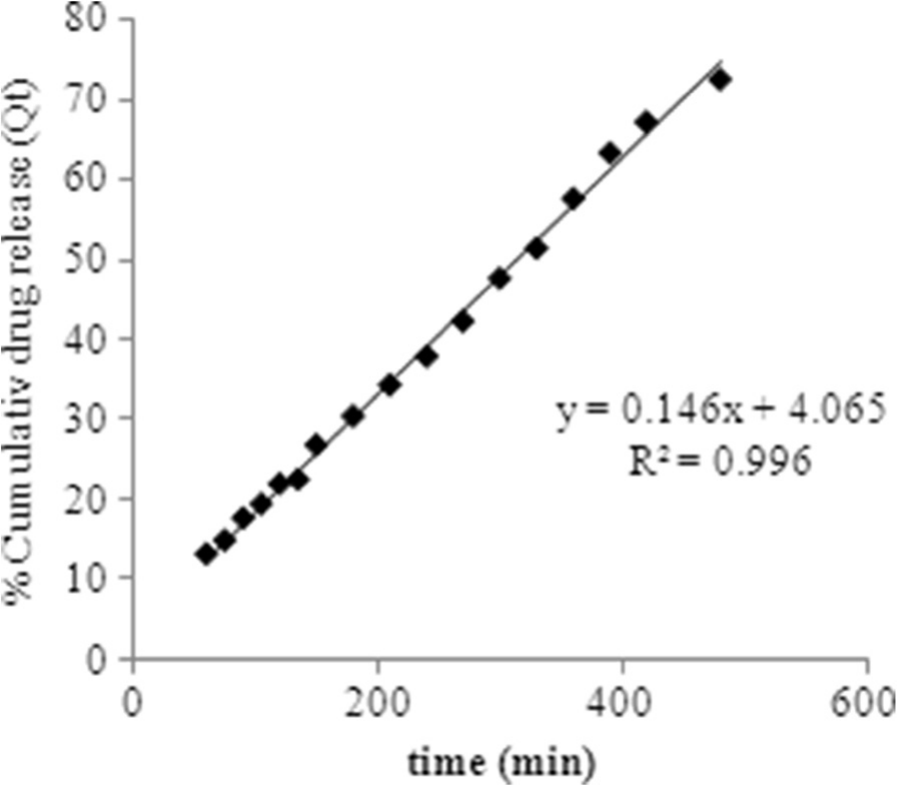
Zero order release kinetics of optimized formulaion having 3.5 mol% EGDMA at pH 8, having drug concentration of 3.2 mg/mL.

**Figure 10:**
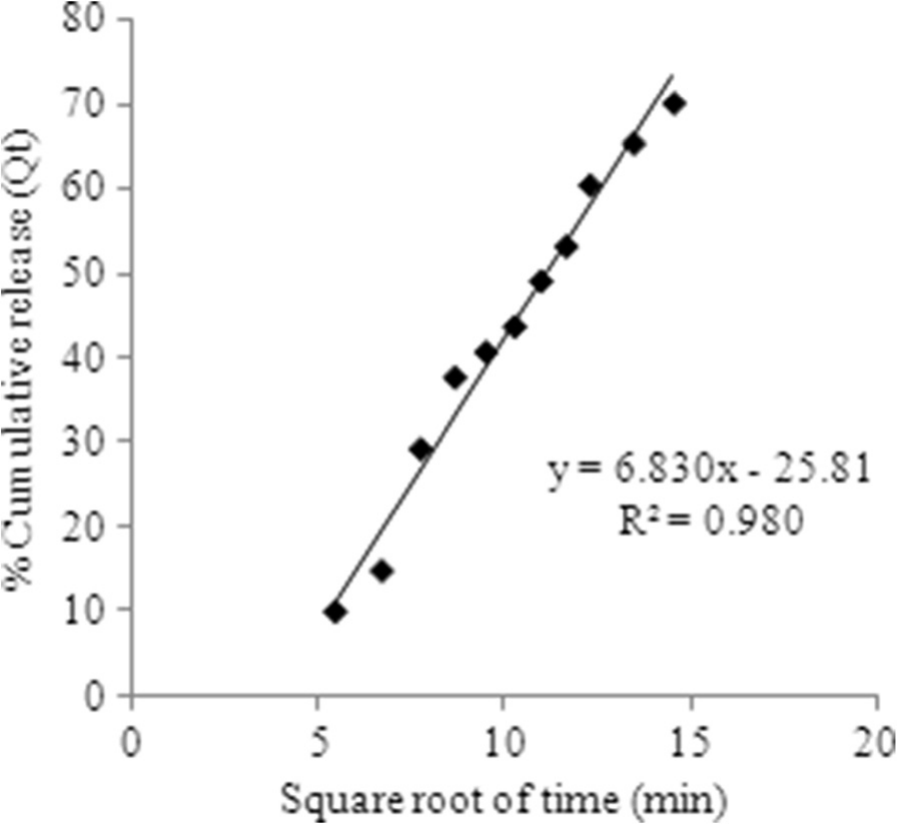
**Higuchi kinetics of optimized formulation E**_**2**_**having drug concentration 0.8 mg/ mL.**

**Figure 11:**
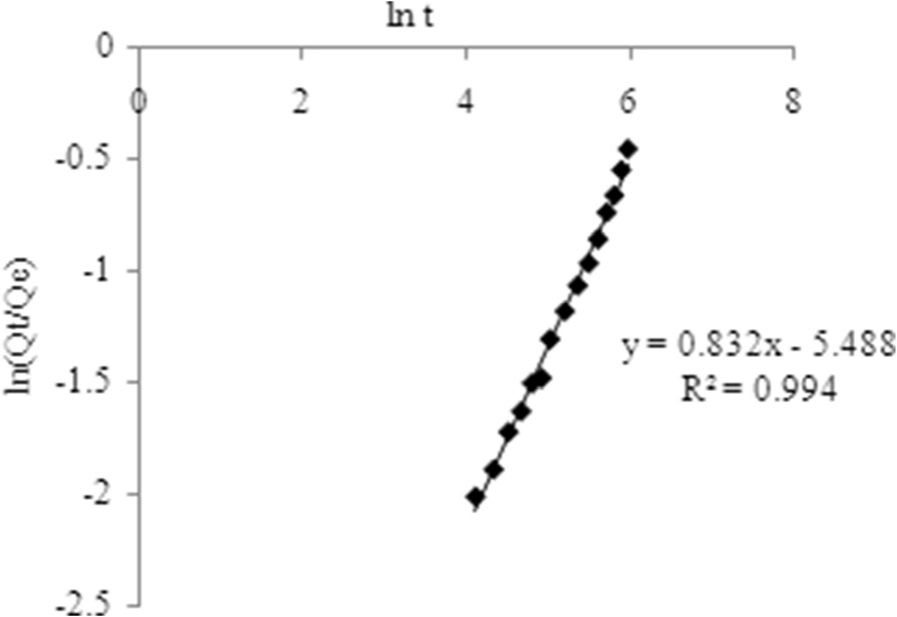
Korsemeyer-Peppas kinetics of optimized formulation having 3.5 mol% EGDMA at pH 8, with a drug concentration of 3.2 mg/mL.

To describe drug release mechanism more precisely, there is a more comprehensive but still very simple semi-empirical formula, called the Korsmeyer-Peppas power law (Eq. 10). So the drug release data were fitted to these kinetic models (Figures 8, 9, 10 and 11) to analyze the release kinetics and the mechanism from the hydrogels. Based on the best correlation coefficient values, the most appropriate model was selected to explain the release behavior of the drug. The values of the release exponent (n), kinetic rate constant (k) and the correlation coefficient (R^2^) are tabulated in the Table 2. Generally speaking, the formulations with varying concentration of the cross linker (E_1_, E_2_ and E_3_) did not seem to obey a zero order kinetics based on the low R^2^ values obtained compared to those of the first order profiles of the drug release. On the other hand the values obtained from other models were found to be very close to each other throughout the whole series of formulations investigated. Nevertheless, with higher concentration of drug loaded, the hydrogels (T_4_, T_5_ and T_6_) were either following the zero order profile or exhibiting very close R^2^ values to those of first order kinetics. It was concluded that these formulations show drug concentration dependency up to a certain limit of drug loaded (T_1_, T_2_ and T_3_). After that threshold concentration of the drug loaded, the release kinetics is observed by other factors like cross link density, chain relaxation and osmotic pressure. Applicability of Hixson-Crowell model to the formulations (T_4_, T_5_ and T_6_) indicated a change in surface area and diameter of the tablets with a progressive dissolution of matrix as a function of time [31]. This result was similar to that obtained when the release behavior of diltiazem HCl from matrix tablets was analyzed by Crohel et al. [32].

The values of “n” determined for chemically cross linked hydrogels studied, ranged from 0.688 to 0.982 as tabulated in the Table 2. The results indicated that all the formulations exhibited anomalous transport (i.e non-Fickian diffusion mechanism), so the drug release was governed by both diffusion of the drug and dissolution of the polymeric network. In fact all the tablets started to erode during the first two hours of their introduction into a fixed volume of phosphate buffer solutions. Even the samples with higher drug concentrations were collapsed during the first hour of exposure of the formulations to the dissolution medium.

### Mechanical strength

The mechanical strength of the xerogels was determined, applying a maximum weight on them. Surprisingly, not a single disk was broken down even the maximum force was applied on them. However, the polymers with low concentration of the cross linker showed a little strain in them by equal increase in their diameter, thus decreasing their thickness; but the observed strain was vanished away within a few minutes after the applied stress was released. The fully swollen hydrogels up to their equilibrium point, exhibited a regular increase in the mechanical strength with the concentration of the cross linker (Table 1). Similar effect of the cross linker on the mechanical strength was also observed by other authors [20].

### DSC and TGA

**Figure 12:**
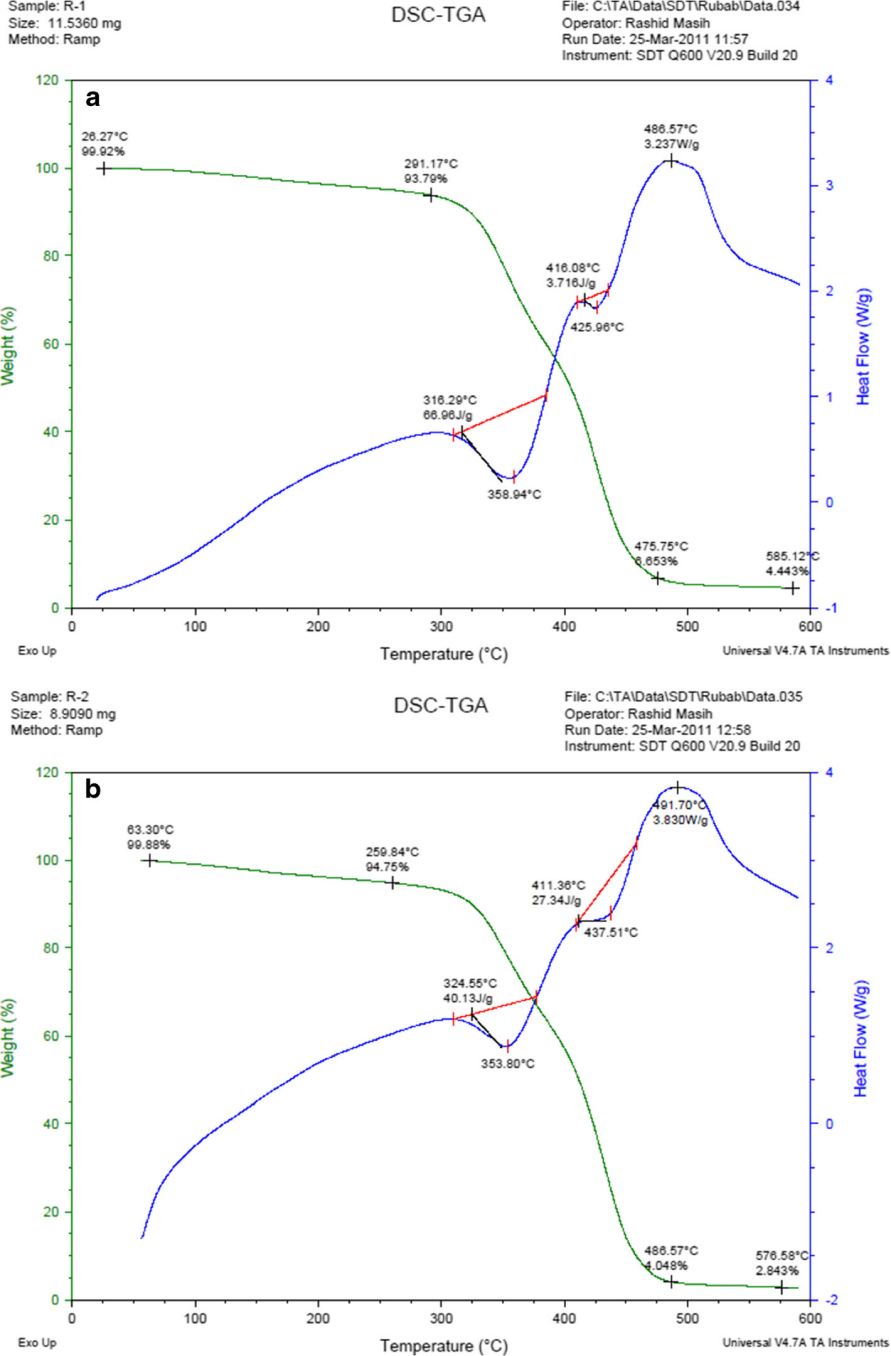
**a.DSC/TGA for E1.b**. DSC/TGA for E2.

In Figure 12, the DSC/TGA curves of the two samples E_1_ and E_2_ before swelling are observed. It is observed that T_g_ of both the samples are not appeared in the thermo gram. If we compare both the samples, it is clear that a rapid increase occurs in heat flow in E_1_ having low mol percent of the cross linker. In TGA curve three steps with difference in weight loss at respective temperatures are observable. The first step in both samples is almost similar showing the presence of almost equal amount of un-reacted volatile monomers as well solvent trapped inside the polymer network. Moreover, a reasonable quantity of moisture may also be present which is removed in the first step. After the removal of moisture and other volatile materials from the samples, side chain decomposition takes place in the range of 300-3.75°C and after that up to 475°C, the back bone degradation is observed. It is clear that most of the weight loss occurred in the second step in a narrow range of temperature (from 300-475°C). After 475°C, up to 600°C, round about, horizontal curve is exhibited by both samples. At the end higher percentage of residue was left behind in E_1_ as compared to E_2_. Exothermic peak after 485°C, in both the samples, indicates phase change taking place in the residual material. The overall analysis indicates that the concentration of the cross linker has no significant effect on thermo gravimetric behavior of these specific systems.

## Conclusions

The chemically cross linked hydrogel copolymer comprised of VA-co-MA-co-AA and EGDMA has proven to be effective controlling the desorption rate of the drug substance from its matrix. The introduction of the higher cross link density decreased (1) the media penetration velocity through the hydrogels in basic media (2) the equilibrium media content in the hydrogels (3) the absorbency of the drug into the polymeric material and (4) the drug release rate from the hydrogels. The shift of the mechanism from diffusion controlled to an anomalous transport changing the pH of the medium from acidic to basic conditions and the mechanical strength indicate that the polymer matrix not only can maintain its structure in the acidic medium of the stomach but also can resist peristaltic movements of the digestive tract, thus preventing the drug release until the target has been achieved. The ability of these ter-polymeric hydrogels systems to load and deliver a drug substance at a controlled rate suggests that these hydrogels show a great promise as a drug delivery system.

## Competing interests

The authors declare that they have no competing interests.

## Authors’ contribution

The main purpose of this paper is to study the drug release behavior of ternary copolymer poly (AAm-co-MA-co-AA). Novelty of the work is Interpretation of media penetration velocity at different pH the media. The relationship of media penetration velocity with equilibrium media sorbed. The effect of crosslinker concentration on above parameters and mechanism of the diffusion. The effect of cross linker concentration on the drug release rate of a model drug. The effect of drug concentration on release behavior. Mechanical strength. DSC/TGA analysis. Application various models to drug release rates. All authors have read and approved this version of the article, and no part of this paper has been published nor is it submitted for publication elsewhere and will not be submitted elsewhere. All authors read and approved the final manuscript.
